# Community-Level Screening and Referral Guidelines for Major Non-Communicable Diseases at Primary Healthcare Settings: From Development to Application at the Multi-site ‘NCDs Mega-Campaign 2024’ in Kathmandu Metropolitan City, Nepal

**DOI:** 10.31729/jnma.8955

**Published:** 2025-04-30

**Authors:** Milan Malla, Richa Nepal, Sushant Regmi, Deewash Neupane, Dhanendra Shrestha, Saugat Bhandari, Madhusudan Subedi

**Affiliations:** 1 Division of Pulmonary Medicine, Mayo Clinic, Florida, USA; 2 Diabetes and Endocrinology Unit, Department of Internal Medicine Medicine, Bir Hospital, Kathmandu, Nepal; 3 Department of General Practice and Emergency Medicine, Patan Academy of Health Sciences, Lagankhel, Lalitpur, Nepal; 4 Patan Academy of Health Sciences, Lagankhel, Lalitpur, Nepal; 5 Department of Community Health Sciences, Patan Academy of Health Sciences, Lagankhel, Lalitpur, Nepal

**Keywords:** *chronic kidney disease*, *diabetes*, *hypertension*, *NCD*, *screening*

## Abstract

Non-communicable diseases (NCDs), mainly cardiovascular conditions like hypertension, diabetes, and chronic kidney disease (CKD) are the major causes of morbidities and mortalities worldwide, with lower- and middle-income countries (LMICs) bearing the highest burden. As the UN 25*25 targets near their final year and the Sustainable Development Goals approach their last trimester, NCD cases continue to rise, leaving a significant undiagnosed population submerged in the communities and households. In Nepal, a few initiatives have been made at the health-facility level over the past decade. However, they lack people-centric strategies and community-focused intervention with appropriate working guidelines. This article outlines the three-phase development of a community-level screening program in Kathmandu Metropolitan City, providing structured guidelines for health workers to screen and appropriately refer cases of hypertension, diabetes, and CKD, implemented during the large-scale community-based campaigns across 256 sites in Kathmandu Metropolitan in 2024.

## INTRODUCTION

Non-communicable diseases (NCDs), such as heart disease, stroke, cancer, diabetes, and chronic lung disease, cause 74% of global deaths. Over three-quarters of all NCDs deaths, including 86% of the 17 million premature deaths (before age 70), occur in low- and middle-income countries (LMICs).^1^ Cardiovascular diseases (CVDs) are the leading cause, with 19 million deaths in 2021, followed by cancers (10 million), chronic respiratory diseases (4 million), and diabetes (over 2 million). These four groups account for 80% of premature NCD deaths. Effective detection, screening, treatment, and palliative care are essential in combating NCDs.^1^

The LMICs often lack primary healthcare programs for early detection and treatment of cardiovascular diseases, leading to late diagnoses and higher mortality at a younger age.^2^ In 2011, the United Nations (UN) Political Declaration on NCDs Prevention and Control set a target to reduce premature mortality from major NCDs by 25% by 2025 (the 25x25 goal). Whereas, Sustainable Development Goals (SDG) 3.4 targets to reduce it by one-third by 2030 to that of 2015.^3^ However, a decade later of the UN political declaration, the global reduction was only 1.5%, highlighting the need for stronger healthcare interventions and equitable access to early detection and treatment.^4^

The 2019 STEP survey reported a 24.5% prevalence of hypertension and 5.8% for diabetes, both showing an increase from 2013 by 1.1% and 2.2% respectively. Alarmingly, 78.8% of hypertensive and 73.5% of diabetic individuals were unaware of their conditions, highlighting the need for frequent community screenings and management plans.^5^ Nepal has implemented intervention guidelines aligned with the Multi-Sectoral Action Plan for the Prevention and Control of NCDs (2014-2020),^6^ introducing the Package of Essential Non-communicable Diseases (PEN) targeting the primary healthcare settings in 2016, with its third edition revised in 2019.^7^

Nepal, like other LMICs, has seen a significant rise in NCD mortality, mainly due to cardiovascular diseases, making it unlikely to meet the 2025 UN targets.^4^ While training and logistical support have improved, community-level interventions remain insufficient. The government lacks clear guidelines for screening and identifying NCD cases at the community level, and their timely referral mechanism which incorporates different levels of government and health facilities.

## DEVELOPING COMMUNITY-BASED SCREENING PROGRAMS AT KATHMANDU METROPOLITAN CITY

To address gaps in early detection, timely management, and referral of major non-communicable diseases at the local level in Nepal, we envisioned community-focused strategies in 2024. The executive board of Kathmandu Metropolitan City (KMC) prioritized these interventions by approving its Health Department's program and budget for the fiscal year 2081/82 BS (2024-25 AD). A unique approach was adopted by designating each Nepali calendar month to a specific major disease for annual screening and awareness campaigns. Bhadra month (August-September) was dedicated to hypertension, Asoj (September-October) to diabetes, Kartik (October-November) to Healthy Diets and Physical Activity, and Mangsir (November-December) to chronic kidney disease (CKD), ensuring a structured and continuous focus on NCDs detection, prevention, and control.

**A. Planning and Preparation phase:**

**Figure 1:**
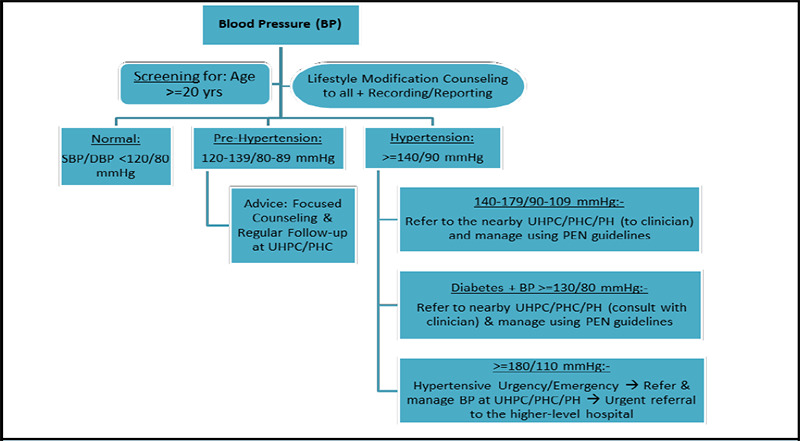
Hypertension screening and referral guidelines at community-level campaigns

**Figure 2:**
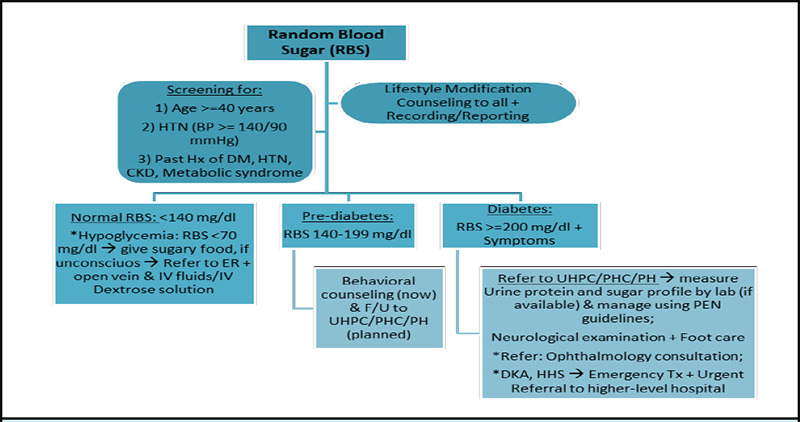
Diabetes screening and referral guidelines at community-level campaigns

**Figure 3:**
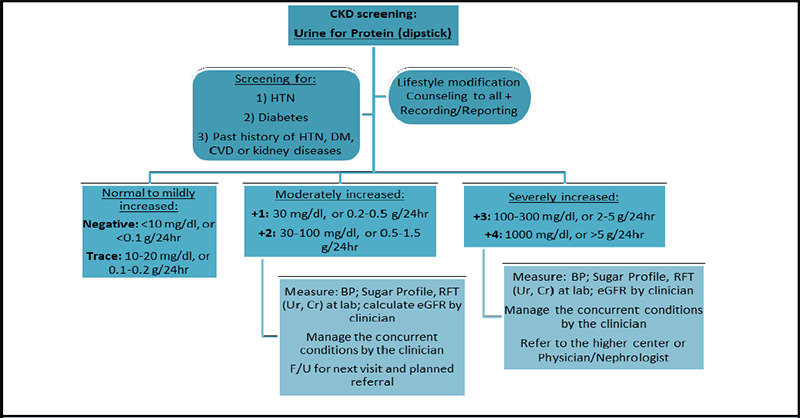
Urine for Protein by dipstick (for CKD) screening and referral guidelines at community-level campaigns

A team of clinical, public health, and managerial experts at the Health Department of Kathmandu Metropolitan City collaborated over several months from Jesth to Bhadra, 2081 (May-September, 2024) to develop comprehensive screening guidelines [Figure 1-3], referral mechanisms, management protocols, training packages, and reporting tools for NCD care. These were refined through multiple workshops and meetings, drawing insights from national training guidelines such as the Package of Essential Non-Communicable Disease Interventions (PEN)7 and international protocols from the American College of Cardiology (ACC)/American Heart Association (AHA), European Society of Cardiology (ESC), Eighth Joint National Committee (JNC-8), American Diabetes Association (ADA), and Kidney Disease Improving Global Outcomes (KDIGO).^8-12^ The developed guidelines may also be useful to other local governments and primary healthcare settings in Nepal and other similar LMICs.

**B. Training and Resource Management phase:**

Primary-level health workers with prior 4-day PEN training from all 32 wards’ Urban Health Promotion Centers (UHPCs) and Primary Health Center (PHC) were selected as the core workforce and health facility ambassadors for the ‘NCDs Mega-Campaign 2024’. They were further trained on the campaign's screening guidelines, covering both theoretical knowledge and practical skills. Screening protocols included hypertension and diabetes assessments using a standardized digital instrument and chronic kidney disease (CKD) screening via urine protein estimation with the dipstick method, followed by appropriate referrals. Additionally, paper-based recording tools for campaign sites and online reporting systems for health facilities were developed and integrated into the training.

The trained health workers conducted further orientation for additional health workers and female community health volunteers (FCHVs) at their respective facilities and wards. The Health Department and ward offices disseminated information about the mega-campaign using various tools and digital media. To ensure efficient execution and achieve a target of a minimum of 50,000 screenings altogether in two months, the Health Department managed all necessary logistics and resources, appointing focal persons and awareness month ambassadors for effective coordination.

**C. Execution and Community Intervention phase:**

During Mangsir and Poush of 2081 (November-December, 2024), each health centre across all 32 wards organized the largest ‘NCDs Mega-Campaign’ to date, conducting screenings and awareness programs at pre-planned eight most-populated locations per ward. This resulted in a total of 256 community sites across Kathmandu Metropolitan City, where 26,019 residents and non-residents actively participated, leading to 47,792 screenings. Screenings followed the developed guidelines at the campaigns [Figure 1-3], while behaviour modification counselling on heart healthy diets, salt and fats reduction, exercise, cessation of smoking and alcohol, treatment adherence, etc. was conducted using pre-developed posters, banners, and audio-visual methods.

Cases requiring further evaluation were referred to respective health centres or hospitals for definitive diagnosis and medical management as per Nepal's PEN protocol. They were initially recorded on paper at screening sites and then entered electronically at health centers the following day with their details. This process enabled the Health Department to systematically identify, register, and quantify cases, facilitating better planning for the continuity of care and long-term management of these chronic NCDs.

## PROPOSED AND APPLIED GUIDELINES AT THE COMMUNITY-LEVEL CAMPAIGNS:

**A. Hypertension screening:**

While ACC/AHA (2017) has already changed the definition of hypertension to >= 130/80 mmHg,^8^ we have been following here the widely applied criteria based on other guidelines such as PEN (2019),^7^ ESC (2024),^9^ and JNC-8 (2014),^10^ as SBP >140 and/or, DBP >=90 mmHg using validated devices and standard measurement protocols. It has been targeted at adults with age >=20 years. For the elevated cases, measurements are taken twice at 5-10-minute intervals. If elevated twice among the three readings, they are referred to the ward's UHPC, PHC, or a primary hospital for further evaluation and management by a medical doctor. Whereas, individuals with diabetes or high-risk CVD history are referred if treatment target thresholds BP ≥130/80 mmHg, based on ACC/AHA (2017).^8^ Hypertensive urgency or emergencies are immediately referred to these centers for acute management and further directed to a higher-level hospital if needed (Figure 1).

**B. Diabetes screening:**

This campaign follows ADA (2024) guidelines for random blood sugar measurements using validated glucometers. While recent ADA update recommends screening at ≥35 years, 11 considering the available workforce at the campaigns, we have set the age at ≥40, including younger individuals with risk factors (Figure 2). Considering the global rise in diabetes trend, future campaigns may lower the age criteria to 30 years as per PEN protocol if resources allow.^7^ Management and referral pathways for hypo-, normal-, and hyperglycemia are detailed in (Figure 2).

**C. Urine protein by dipstick for CKD screening:**

Early detection and treatment of CKD in targeted populations in the community setting is beneficial to reduce the incidence of both CVD and CKD progression to kidney failure through earlier interventions.^12,13^ Hypertension, diabetes, or histories of CVD (e.g. heart failure) or kidney diseases were the primary target groups for the screening. The KDIGO guideline has recommended testing people at risk for and with chronic kidney disease using urine albumin (albumin-creatinine ratio ‘ACR') measurement and estimated glomerular filtration rate (eGFR).^12^ We used urine protein by dipstick method, another suggested semi-quantitative first-line and feasible method for screening and initial urine protein level categorization followed by renal function test (urea, creatinine) at the health facility if the dipstick shows +1 or higher gradings (Figure 3).

## CONCLUSION AND WAY FORWARD:

Early detection, timely interventions, and preventive initiatives are key for minimizing the huge economic, societal, and healthcare burden of NCDs, particularly in the LMICs. Currently, only the tip of the iceberg of NCD cases is being identified at the health facility level. First, an active nationwide surveillance strategy, such as mass screening campaigns at regular intervals at the community (ward or tole) level, should be initiated. This approach, centered on a people-centric healthcare model, is necessary to replace the traditional passive surveillance system confined to health facilities.

Second, screening should never be conducted merely for the sake of it. A well-structured delivery mechanism — before, during, and after the campaigns— is essential. This includes setting feasible goals, predefined and evidence-based screening guidelines, allocating adequate resources, using validated tools, building the capacity of healthcare workers, and ensuring their guided mobilization along with research and evidence-generating recording system. Most importantly, post-screening management plans must be in place, with a clearly defined referral mechanism and strong linkages to the health facilities, ensuring minimal or no burden on the clients.

Third, achieving the UN's 2025 targets seems unlikely for Nepal, and meeting the SDG-3.4 by 2030 looks profoundly challenging. To reduce the burden and mortality of NCDs, all levels of government must urgently gear up their community- and people-centric innovations and strategies. These efforts will be a cornerstone in addressing the growing NCD crisis over the next decade. These applied guidelines and insights from the community-level NCDs mega-campaigns conducted in Kathmandu Metropolitan City in 2024 may serve as a valuable reference for executing similar initiatives at different levels in the future.
